# Knockdown of DDX46 inhibits trophoblast cell proliferation and migration through the PI3K/Akt/mTOR signaling pathway in preeclampsia

**DOI:** 10.1515/biol-2020-0043

**Published:** 2020-06-13

**Authors:** Xin You, Hongyan Cui, Ning Yu, Qiuli Li

**Affiliations:** Department of Obstetrics, Tianjin Central Hospital of Gynecology Obstetrics, Tianjin 300052, China; Department of Laboratory, Tianjin Central Hospital of Gynecology Obstetrics, Tianjin 300052, China

**Keywords:** preeclampsia, trophoblast cells, RNA helicases, DDX46, PI3K/Akt/mTOR pathway

## Abstract

Preeclampsia (PE) is a serious disease during pregnancy associated with the dysfunction of trophoblast cell invasion. DDX46 is a kind of RNA helicase that has been found to regulate cancer cell metastasis. However, the role of DDX46 in PE remains unclear. Our results showed that the mRNA levels of DDX46 in placental tissues of pregnant women with PE were markedly lower than those in normal pregnancies. Loss-of-function assays showed that knockdown of DDX46 significantly suppressed cell proliferation of trophoblast cells. Besides, DDX46 knockdown decreased trophoblast cell migration and invasion capacity. In contrast, the overexpression of DDX46 promoted the migration and invasion of trophoblast cells. Furthermore, knockdown of DDX46 caused significant decrease in the levels of p-PI3K, p-Akt, and p-mTOR in HTR-8/SVneo cells. In addition, treatment with IGF-1 reversed the inhibitory effects of DDX46 knockdown on proliferation, migration, and invasion of HTR-8/SVneo cells. In conclusion, these data suggest that DDX46 might be involved in the progression of PE, which might be attributed to the regulation of PI3K/Akt/mTOR signaling pathway. Thus, DDX46 might serve as a therapeutic target for the treatment of PE.

## 1 Introduction

Preeclampsia (PE) is a serious disease during pregnancy (after the twentieth week of gestation) [1]. PE is characterized by hypertension ≥140 mmHg on two separate occasions (≥4 h apart) and proteinuria ≥0.3 g in urine [2]. It has been conveyed that PE accounts for 2–8% of pregnancies, and it is responsible for approximately 15% of maternal morbidity and mortality [3,4]. Therefore, developing effective therapies for the prevention and treatment of PE is a hot issue in the field of reproductive medicine.

The pathogenesis of PE is complex and affected by numerous genetic, immunologic, and environmental factors [5,6,7]. Currently, it is generally believed that dysfunction of trophoblast invasion into the endometrium occurring in the early stage of pregnancy may lead to the uterine spiral artery remodeling disorder, which subsequently contributes to the development of PE [8,9,10]. Therefore, the impaired trophoblast invasion is considered as the main cause of PE.

RNA helicases are a large group of enzymes that participate in all biochemical steps involving RNA processing, including splicing, transcription, translation, transport, decay, and ribosome biogenesis [11,12,13]. Therefore, RNA helicases are involved in several kinds of diseases, such as infections and cancers [14,15]. The DEAD-box (DDX) helicases are crucial RNA helicases that are highly conserved from bacteria to humans [16]. DDX46 is a member of DDX family and has been found to exert various processes, such as tumorigenesis, antiviral effect, and development of digestive organs and brain [17,18,19,20,21,22,23]. However, the function of DDX46 in PE remains unclear.

In the current study, we investigated the role of DDX46 in trophoblast cell proliferation and invasion. The results showed that knockdown of DDX46 suppressed cell proliferation, migration, and invasion in trophoblast cells.

## 2 Materials and methods

### 2.1 Tissue specimens

Twenty-five pregnant women with PE and 25 normal pregnancies who had experienced cesarean deliveries at Tianjin Central Hospital of Gynecology Obstetrics from March 2017 to April 2018 were enrolled in the present study. The placental tissues (1.0 × 1.0 × 1.0 cm) were collected within 5 min after the separation of placenta.


**Informed consent:** Informed consent has been obtained from all individuals included in this study.
**Ethical approval:** The research related to human use has been complied with all the relevant national regulations, institutional policies and in accordance with the tenets of the Helsinki Declaration, and has been approved by the Ethics Committee of Tianjin Central Hospital of Gynecology Obstetrics.

### 2.2 Cell culture and treatment

Human trophoblast cell lines JEG3 and HTR-8/SVneo cells (American Type Culture Collection company, Manassas, VA, USA) were cultured in the DMEM/F12 medium containing 10% fetal bovine serum (FBS) and double antibiotics (100 µg/mL streptomycin and 100 UI/mL penicillin). The cells were maintained in an incubator with 5% CO_2_ at 37°C until the growth density reached 80–90%. In order to explore the underlying molecular mechanism, JEG3 cells were transfected with si1-DDX46 or si-NC in the presence or absence of IGF-1 (10 µM) for 48 h. Cell proliferation, migration, and invasion were detected.

### 2.3 Cell transfection

Two specific siRNAs targeting DDX46 (si1-DDX46 and si2-DDX46) and the scrambled negative control (si-NC) were purchased from GenePharm (Shanghai, China). For siRNA transfection, trophoblast cells were transiently transfected with indicated siRNAs (100 nM) using Lipofectamine 2000 (Invitrogen, Carlsbad, CA, USA) following the manufacturer’s instruction.

The full-length DDX46 cDNA was amplified and sub-cloned into pcDNA3.1 (Invitrogen), whereas the empty vector pcDNA3.1 was used as control. Trophoblast cells were transfected with pcDNA3.1-DDX46 or pcDNA3.1 using Lipofectamine 2000 (Invitrogen) according to the manufacturer’s instructions.

### 2.4 Quantitative real-time PCR (qRT-PCR)

The total RNA was extracted using TRIzol reagent (Invitrogen), and the RNA concentration was measured. Then, the RNA was reverse transcribed into cDNA using PrimeScript RT Master Mix (Takara, Dalian, China) according to the instructions, followed by PCR amplification using the SYBR Premix Ex Taq II Kit (Takara). The PCR amplification program was carried out under the following conditions: an initial denaturation step, 1 min at 94°C, followed by 40 cycles, 95°C for 30 s, and 60°C for 1 min. Primers were listed as follows: DDX46 forward, 5′-AAA ATG GCG AGA AGA GCA ACG-3′ and reverse, 5′-CAT CAT CGT CCT CTA AAC TCC AC-3′; β-actin forward, 5′-ATC ACC ATT GGC AAT GAG CG-3′, reverse 5′-TTG AAG GTA GTT TCG TGG AT-3′.

### 2.5 Western blot

The protein was extracted using RIPA reagent (Beyotime, Shanghai, China), and the protein concentration was detected using BCA Assay Kit (Beyotime). The protein was loaded with 12% sodium dodecyl sulfatepolyacrylamide gel electrophoresis, after that, the protein was transferred onto the polyvinylidene difluoride membrane. Following 2 h blocking in 5% skimmed milk, the membrane was incubated with the primary antibodies against p-PI3K, PI3K, Akt, p-Akt, mTOR, p-mTOR, and β-actin (diluted in 1:500; Abcam, Cambridge, MA, USA) at 4°C overnight. On the next day, the protein was incubated with the secondary antibody (diluted in 1:3,000; Abcam) for 1 h at room temperature. The bands were developed using ECL Detection System (Life technologies, Grand Island, NY, USA).

### 2.6 Cell proliferation assay

Cells in the logarithmic growth phase were seeded into a 96-well plate at a density of 1 × 10^3^ cells/well and cultured in an incubator overnight. After that, 10 µL of CCK-8 solution was added into the well, followed by an incubation for 4 h. The absorbance (*λ* = 450 nm) was measured using a multifunctional microplate reader (Bio-Tek Instruments, Winooski, VT, USA).

### 2.7 Cell invasion and migration assays

Cell invasion and migration capacity were assessed by transwell assay. The cells were resuspended in serum-free medium and added into the transwell upper chamber, and the cell medium containing 10% FBS was added into the lower chamber. Following an incubation for 24 h, cells on the lower side of the inserts were fixed with 4% paraformaldehyde for 10 min and stained with 0.5% crystal violet for 10 min. Finally, the number of stained cells was counted under a microscope. For the invasion assay, the transwell chambers were coated with Matrigel (BD Bioscience, Franklin Lakes, NJ, USA).

### 2.8 Statistical analysis

GraphPad 5.0 software (GraphPad Software Inc., San Diego, CA, USA) was used for statistical processing of all data. Student’s *t* test was used to assess statistical differences between two groups. One-way analysis of variance was used to assess statistical differences among multiple groups. All data are expressed as mean ± standard deviation. The *p* < 0.05 suggested that the difference was statistically significant.

## 3 Results

### 3.1 DDX46 was lowly expressed in the placentas of patients with PE

**Figure 1:**
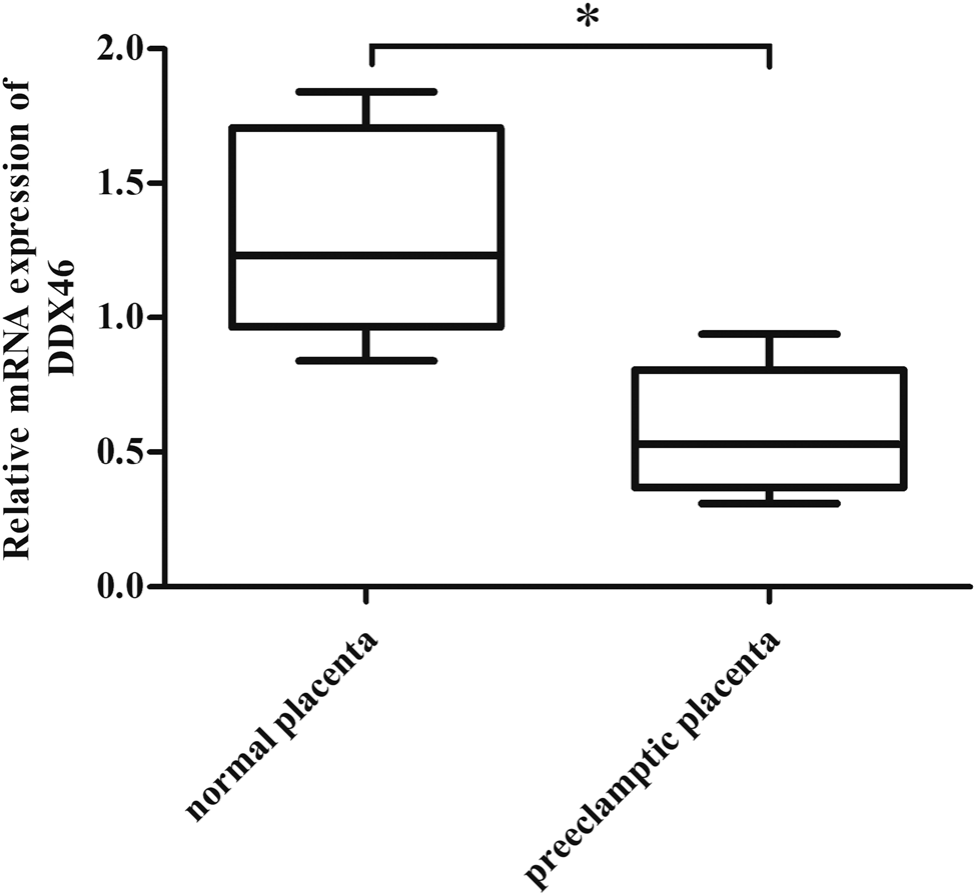
The mRNA levels of DDX46 in the placentas of patients with PE and normal pregnancies. The qRT-PCR was performed to evaluate the mRNA levels of DDX46 in placental tissues of pregnant women with PE (*n* = 25) and normal pregnancies (*n* = 25). *p < 0.05 vs normal pregnancies.

First, the mRNA levels of DDX46 in placental tissues of pregnant women with PE (*n* = 25) and normal pregnancies (*n* = 25) were evaluated. The results of qRT-PCR revealed that the DDX46 gene expression in pregnant women with PE was significantly lower than that in normal pregnancies (Figure 1).

### 3.2 Knockdown of DDX46 inhibited trophoblast cell proliferation

**Figure 2:**
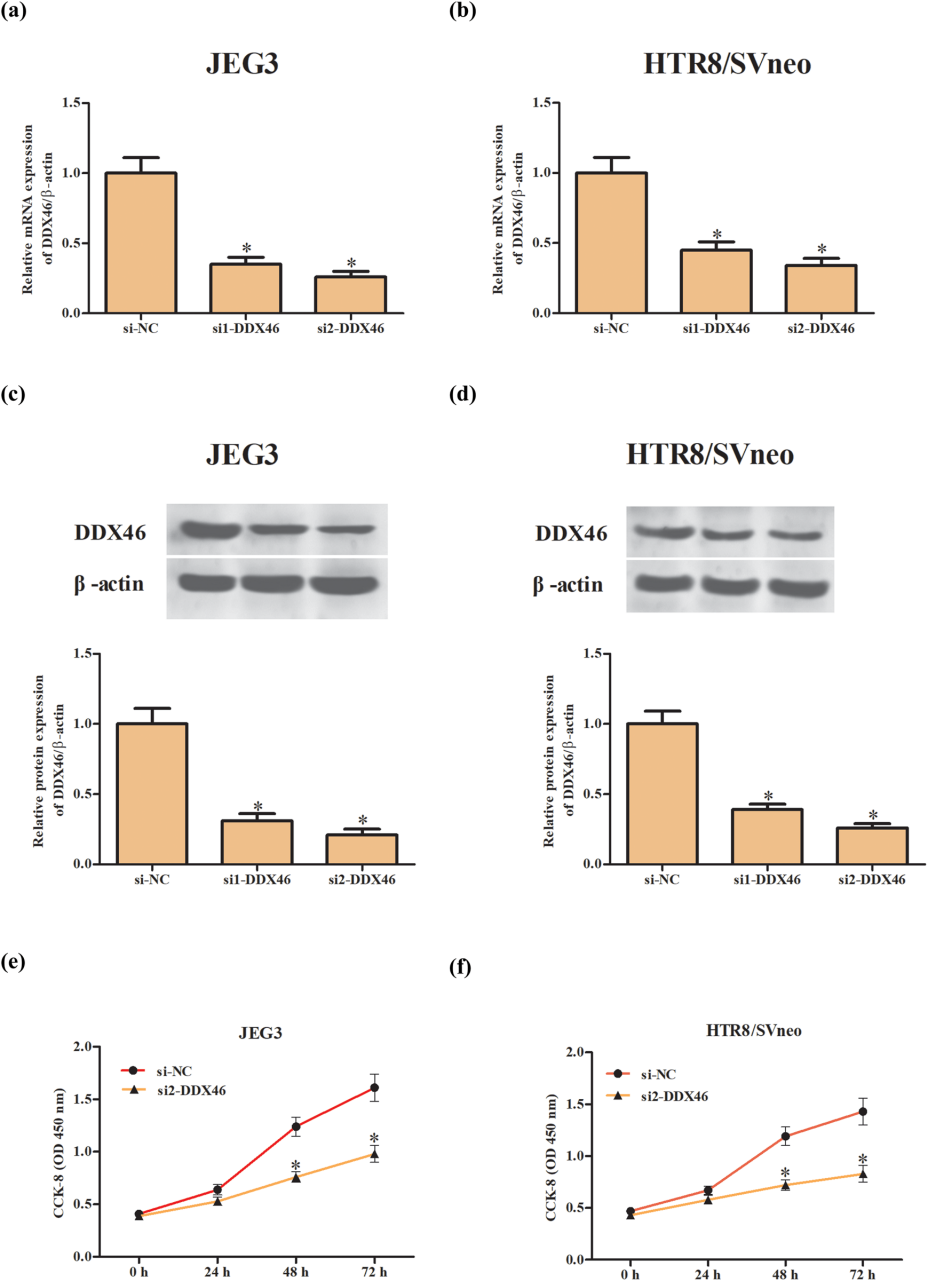
Effect of DDX46 knockdown on the proliferation of trophoblast cells. (a and b) The mRNA levels of DDX46 in JEG3 and HTR-8/SVneo cells transfected with si1-DDX46 or si2-DDX46 or si-NC. (c and d) The protein expression levels of DDX46 in JEG3 and HTR-8/SVneo cells transfected with si1-DDX46 or si2-DDX46 or si-NC. (e and f) CCK-8 assay was performed to detect the cell proliferation of JEG3 and HTR-8/SVneo cells. *p < 0.05.

To further explore the biological function of DDX46 in trophoblast cells, the JEG3 and HTR-8/SVneo cells were transfected with first siRNA sequence against DDX-46 (si1-DDX46) or second siRNA sequence against DDX-46 (si2-DDX46) or negative control siRNA (si-NC). The results revealed that the mRNA and protein expression levels of DDX46 in si1-DDX46 or si2-DDX46 transfected JEG3 cells were significantly decreased when compared with si-NC transfected JEG3 cells. The knockdown efficiency of si2-DDX46 was significantly higher than si1-DDX46 (Figure 2a and c). Meanwhile, the expression levels of DDX46 were markedly reduced in HTR-8/SVneo cells after transfection with si1-DDX46 or si2-DDX46. Besides, transfection with si2-DDX46 exhibited stronger knockdown efficiency than si1-DDX46 (Figure 2b and d). Therefore, the si2-DDX46 was used for the inhibition of DDX46 in JEG3 and HTR-8/SVneo cells. The following CCK-8 assay proved that knockdown of DDX46 suppressed the cell proliferation of JEG3 and HTR-8/SVneo cells (Figure 2e and f).

### 3.3 Knockdown of DDX46 inhibited the migration and invasion of trophoblast cells

**Figure 3:**
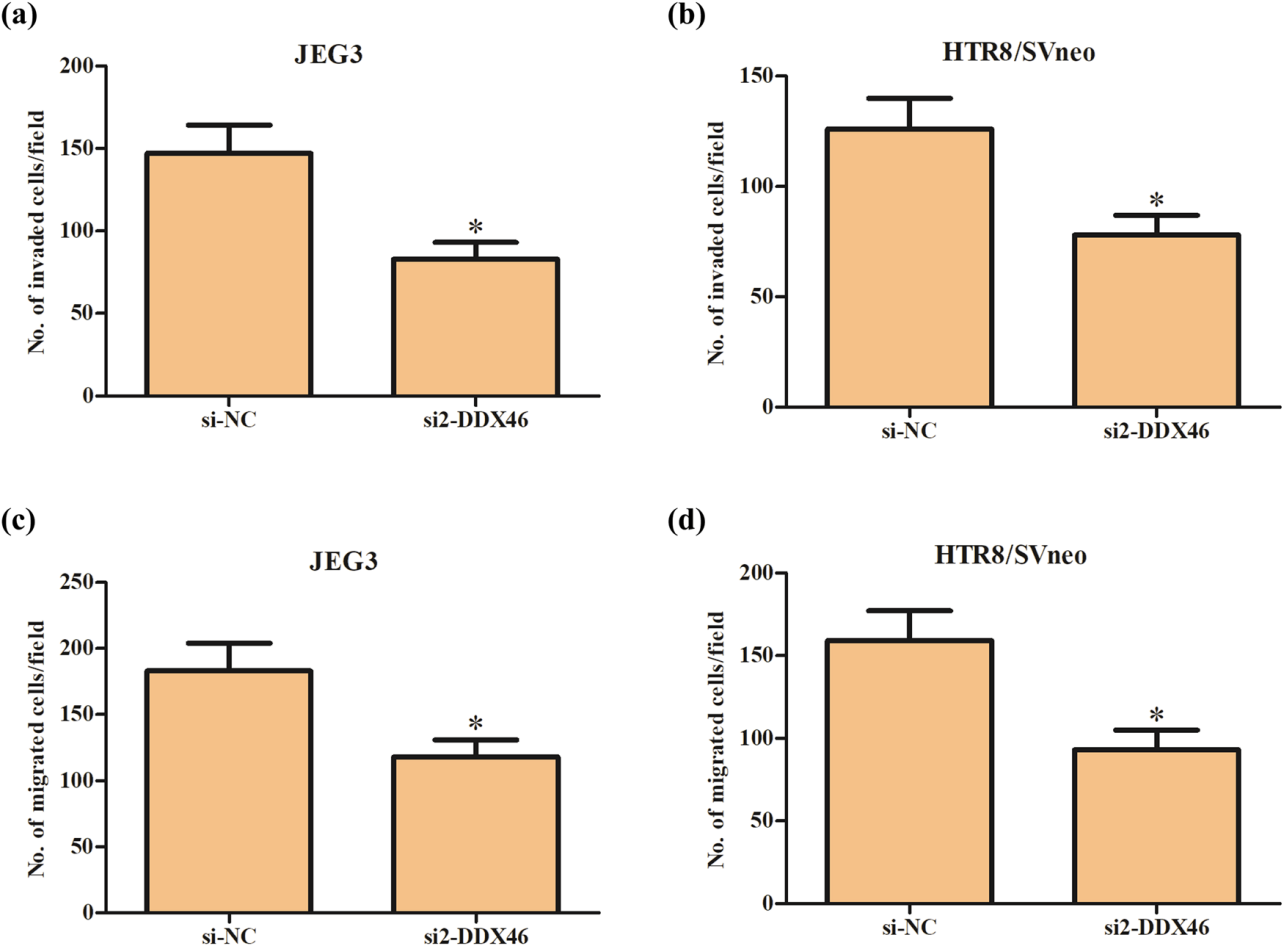
Effect of DDX46 knockdown on migration and invasion of trophoblast cells. Transwell assay was employed to detect the invasion and migration capacity of JEG3 and HTR-8/SVneo cells. (a and b) Invasion capacity of JEG3 and HTR-8/SVneo cells. (c and d) Migration capacity of JEG3 and HTR-8/SVneo cells. *p < 0.05.

In addition, transwell assay was employed to detect the invasion and migration capacity. The data showed that knockdown of DDX46 obviously reduced the invasion capacity of JEG3 and HTR-8/SVneo cells (Figure 3a and b). At the same time, we also found that the migration capacities of JEG3 and HTR-8/SVneo cells were also decreased after transfection with si2-DDX46 (Figure 3c and d).

### 3.4 Overexpression of DDX46 promoted the migration and invasion of trophoblast cells

**Figure 4:**
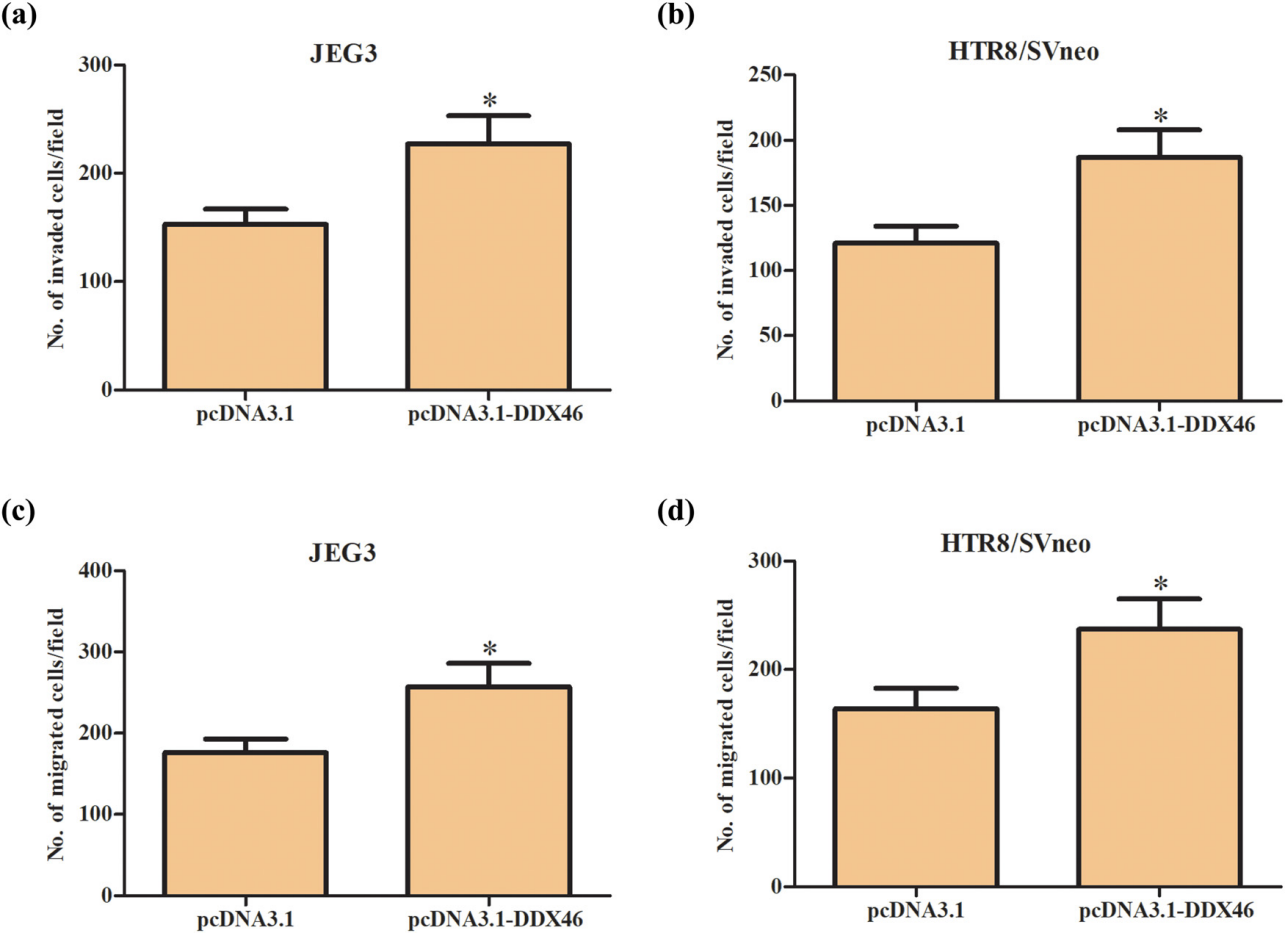
Effect of DDX46 overexpression on migration and invasion of trophoblast cells. Transwell assay was employed to detect the invasion and migration capacity of JEG3 and HTR-8/SVneo cells. (a and b) Invasion capacity of JEG3 and HTR-8/SVneo cells. (c and d) Migration capacity of JEG3 and HTR-8/SVneo cells. *p < 0.05.

Then, we examined the effects of DDX46 overexpression on trophoblast cell migration and invasion. As indicated in Figure 4a and b, overexpression of DDX46 significantly promoted the invasion of JEG3 and HTR-8/SVneo cells, respectively (Figure 4a and b). In addition, the number of migrated cells was greatly increased in JEG3 and HTR-8/SVneo cells transfected with DDX46 overexpression (Figure 4c and d).

### 3.5 Knockdown of DDX46 inhibited the activation of PI3K/Akt/mTOR signaling in trophoblast cells

**Figure 5:**
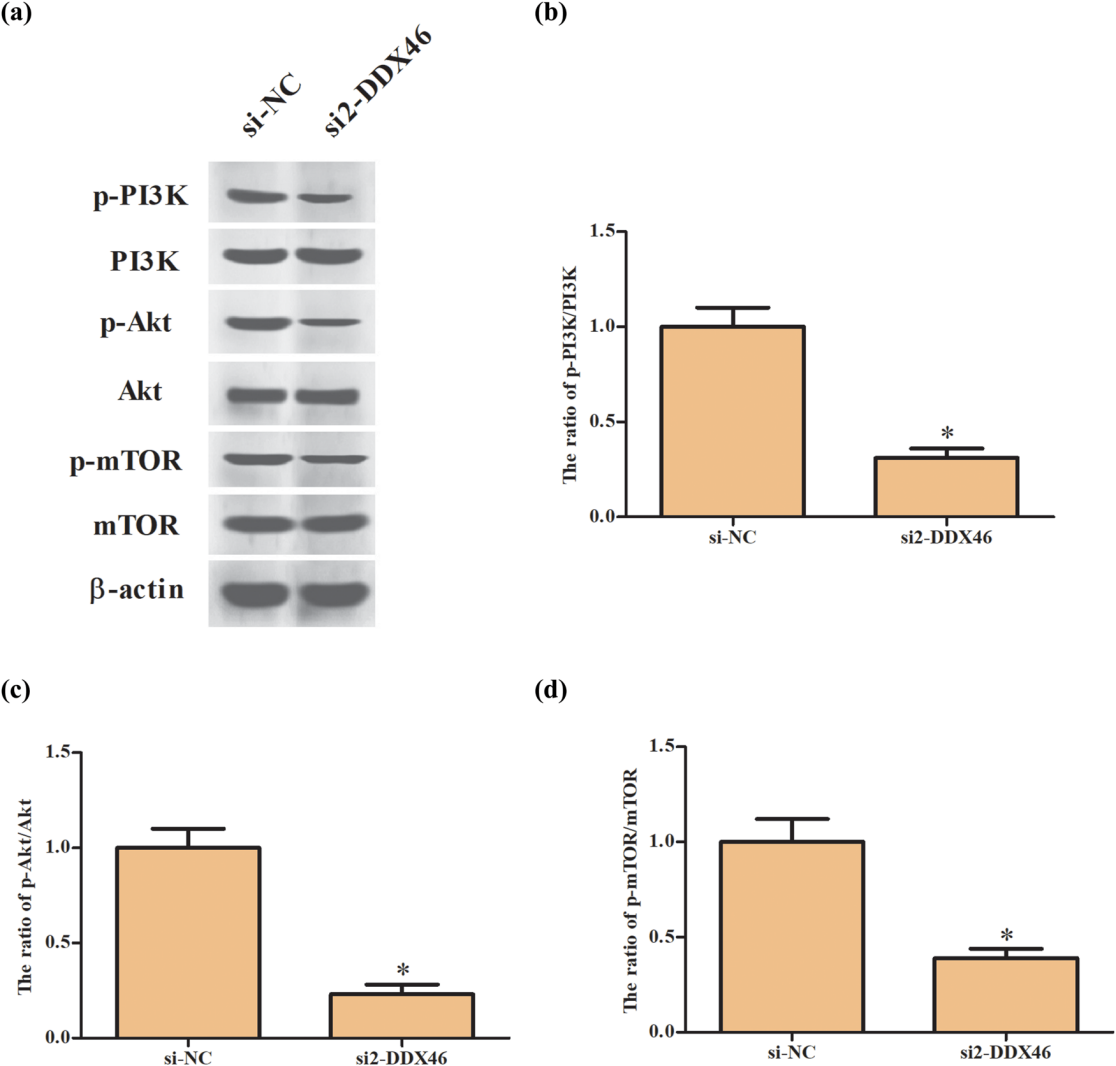
Effect of DDX46 knockdown on PI3K/Akt/mTOR signaling in trophoblast cells. (a) The effect of DDX46 knockdown on PI3K/Akt/mTOR signaling pathway was evaluated by detecting the expression levels of p-PI3K, PI3K, Akt, p-Akt, mTOR, and p-mTOR in JEG3 cells using western blot. (b) The ratio of p-PI3K/PI3K. (c) The ratio of p-Akt/Akt. (d) The ratio of p-mTOR/mTOR. *p < 0.05.

Since the PI3K/Akt/mTOR signaling pathway has been shown to be associated with metastasis of trophoblast cells [24], and DDX46 was reported to regulate the PI3K/Akt pathway in osteosarcoma [19]. Thus, we further explored the effect of DDX46 knockdown on the pathway in trophoblast cells. Western blot analysis showed that the expression levels of p-PI3K, p-Akt, and p-mTOR were significantly decreased in JEG3 cells transfected with si2-DDX46 when compared with JEG3 cells transfected with si-NC (Figure 5).

### 3.6 PI3K/Akt/mTOR pathway activator IGF-1 reversed the effects of DDX46 knockdown on trophoblast cells

**Figure 6:**
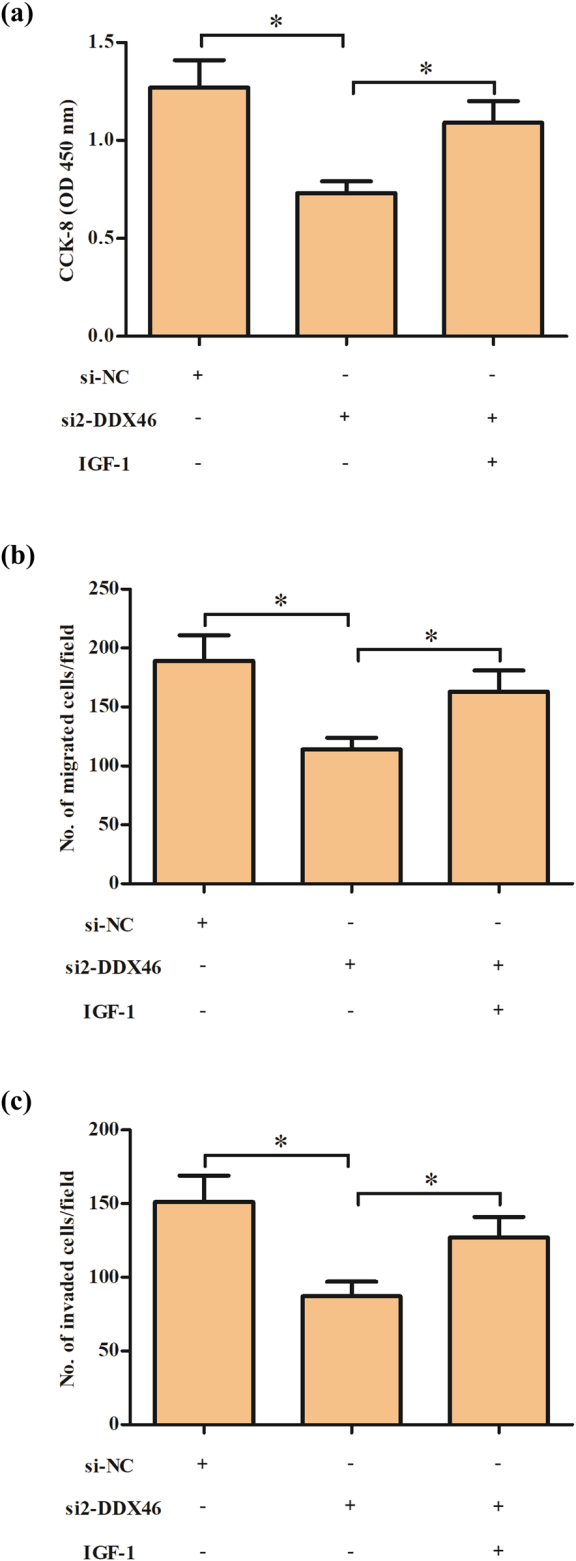
Effect of IGF-1 on the effects of DDX46 knockdown on trophoblast cells. JEG3 cells were treated with IGF-1, which is an activator of PI3K/Akt/mTOR pathway. (a) CCK-8 assay was performed to detect the cell proliferation of JEG3 cells. (b and c) Transwell assay was employed to detect the invasion and migration capacity of JEG3 cells. *p < 0.05.

To further confirm the role of PI3K/Akt/mTOR pathway, JEG3 cells were treated with IGF-1, which is an activator of PI3K/Akt/mTOR pathway. As shown in Figure 6a, treatment with IGF-1 promoted cell proliferation of si2-DDX46 transfected JEG3 cells. In addition, the si2-DDX46-caused reduction in migration and invasion capacity of JEG3 cells was reversed by IGF-1 (Figure 6b and c).

## 4 Discussion

Trophoblast cells, the main components of the placenta, are essential to the formation of the placenta and maintaining the development of normal fetal [25]. Trophoblast cells’ migration and invasion into the maternal endometrium are important for successful implantation and placentation [26,27]. However, dysfunctional trophoblast migration and invasion can lead to the uterine spiral artery remodeling disorder and fetal growth retardation, which subsequently leads to PE [28]. Currently, it is generally believed that impaired trophoblast invasion is the main cause of PE. Inhibition of trophoblast cell proliferation and invasion is crucial for preventing the development of PE. Wang et al. [29] reported that CXCL3 is involved in the migration, invasion, proliferation, and tubule formation of trophoblasts, implying that CXCL3 may play a key role in the pathogenesis of PE. CircHIPK3 is decreased in PE and affects proliferation, migration, invasion, and tube formation of human trophoblast cells [30]. These findings suggested that targeting functional molecules may be an effective therapeutic approach for the prevention and treatment of PE.

DDX46 is a kind of RNA helicase that has been found to regulate cell proliferation and metastasis of cancer cells. Knockdown of DDX46 obviously inhibits osteosarcoma cell proliferation and suppresses migration and invasion in osteosarcoma cells [19]. DDX46 knockdown leads to decreased proliferation in esophageal squamous cell carcinoma cells [20]. Lentiviral DDX46 knockdown inhibits cell proliferation of human colorectal cancer cells [21]. In the current study, we found that the mRNA levels of DDX46 in placental tissues of pregnant women with PE were markedly lower than that in normal pregnancies. Loss-of-function assays showed that knockdown of DDX46 in trophoblast cells significantly suppressed cell proliferation, migration, and invasion. The results indicated that DDX46 might be involved in the progression of PE and might serve as a therapeutic target.

PI3K/Akt/mTOR pathway is an important signaling pathway involved in multiple cellular processes, such as differentiation, proliferation, migration, and invasion [31]. Many investigations have demonstrated that the PI3K/Akt/mTOR pathway is associated with the cell behaviors of trophoblast cells. Wang et al. [32] demonstrated that reduced expression of ELABELA (ELA) attenuates trophoblast invasion through the PI3K/Akt/mTOR pathway in early onset PE. Downregulation of receptor tyrosine kinase-like orphan receptor 1 (ROR1) in PE placenta inhibits human trophoblast cell proliferation, migration, and invasion via regulating the PI3K/Akt/mTOR pathway [24]. Laminin (LN)-α5 downregulation is associated with PE placenta and impairs trophoblast cell viability and invasiveness through the PI3K/Akt/mTOR pathway, which could be a causative factor of PE pathogenesis [33]. Concerning the mechanism of the DDX46 function, we further discovered that knockdown of DDX46 caused significantly decrease in the expression levels of p-Akt and p-mTOR in trophoblast cells. The results implied that DDX46 knockdown inhibited the activation of PI3K/Akt/mTOR signaling pathway in trophoblast cells. Furthermore, treatment with IGF-1 reversed the inhibitory effects of DDX46 knockdown on trophoblast cell proliferation, migration, and invasion. Collectively, the role of DDX46 in PE was mediated by the PI3K/Akt/mTOR signaling pathway.

## 5 Conclusion

In summary, we reported that DDX46 exhibited significant effects on trophoblast cell proliferation, migration, and invasion. Further experiments demonstrated that the inhibitory effects of DDX46 knockdown on cell behaviors of trophoblast cells were regulated by the PI3K/Akt/mTOR signaling pathway. Therefore, our results provided evidence for the treatment of PE.
